# A Surface-Enabled Computational Homogenization Method for Variable-Density Polymer Lattice Metastructures

**DOI:** 10.3390/polym17060769

**Published:** 2025-03-14

**Authors:** Aofei Zhang, Shuo Li, Ling Ling, Li Li

**Affiliations:** State Key Laboratory of Intelligent Manufacturing Equipment and Technology, School of Mechanical Science and Engineering, Huazhong University of Science and Technology, Wuhan 430074, China

**Keywords:** lattice metastructure, polymer, surface-enhanced computational homogenization, metamaterial, surface effect, variable density surface law

## Abstract

The current limitations in predicting mechanical properties arise from an incomplete understanding of surface-induced size effects in variable-density polymer lattice metastructures. Through large-scale, high-fidelity finite element simulations, we identify a novel variable-density surface law governing the surface intrinsic length at the macroscopic scale. Capitalizing on this surface law discovery, we propose a surface-enhanced computational homogenization framework. By incorporating the surface intrinsic length parameters with the variable-density surface law and an offline database constructed through high-throughput numerical simulations, we develop an efficient predictive model capable of online analysis for the mechanical behavior of variable-density polymeric lattice metastructures. This innovative approach preserves critical configuration-dependent surface effects while achieving both efficiency and precision in predicting the macro-scale mechanical performance of such metastructures.

## 1. Introduction

Polymers are widely used in industrial settings due to their lightweight nature, ease of processing, corrosion resistance, electrical insulation, and design flexibility [1,2]. Metamaterials typically refer to artificial structures that obtain novelty extraordinary properties not found in nature through structural engineering rather than chemical composition, such as negative Poisson’s ratio [3,4], non-local characteristics [5,6], negative refractive index [7,8], and surface effects [9]. These novelty extraordinary properties of a polymeric metamaterial should be dependent on its microstructure [10] rather than its chemical composition. Variable-density polymer lattice metastructures (VDPLMs) demonstrate core advantages through spatially graded density design and customizable performance, particularly in lightweight designs [11] and energy absorption. Key applications include the following: (1) aerospace components (e.g., satellite brackets and fairings) requiring lightweight and impact resistance, (2) biomedical devices such as customized orthopedic implants and bionic menisci that employ density gradients to mimic cartilage’s stress transfer mechanisms, and (3) bio-inspired energy-absorbing structures for specialized applications [12,13,14].

Polymer materials have a variety of properties, and these properties are affected by many factors. The lightness of polymers is one of their most important advantages. Lightweight polymer materials usually have low density and a good strength-to-weight ratio, which makes them widely used in aerospace [15,16], automotive [17,18], construction [19,20], and other fields [21]. The lightweight performance of polymers is affected by many factors, mainly their chemical structure, molecular design, fillers and reinforcement materials, processing technology, microstructure, and other aspects. High design flexibility is due to various factors influencing polymers. This makes it easier for researchers to design polymers with excellent properties [22,23,24]. However, the regulation of polymer properties is mostly at the microscopic level, which limits design flexibility.

Lattice metastructures attracted widespread attention in the early 20th century due to their high specific strength, high specific stiffness, vibration, noise reduction [25], and ease of design [26,27,28]. The lattice metastructure is a multifunctional material with interconnected rods, plates, or complex surfaces as the basic unit and a repeated regular arrangement. Due to its complex structure and processing difficulty, the relevant research has remained in the theoretical stage for a long time. The rapid development of additive manufacturing technology [29,30,31] in recent years has provided technical support for the theoretical research and engineering application [32,33,34] of lattice metastructures. Polymer materials have excellent performance, design flexibility [35,36,37], and certain special properties (such as shape memory polymers [38,39] and biodegradable polymers [40,41]) and are one of the materials commonly used in additive manufacturing. Using polymers as the material and a variable-density lattice as the geometry, the VDPLM manufactured by additive manufacturing technology has the advantages of multiple technologies and can design the performance of the structure at multiple scales. Therefore, it is important to calculate the performance of VDPLMs accurately and efficiently.

Compared with the uniform-density lattice metastructure, the VDPLM is more flexible and adaptable [42,43]. This is characterized by the gradual distribution of lattice attributes, which allows the optimal layout to be designed by considering the performance requirements of different positions within the structure. Cheng et al. [44] used a topology optimization algorithm to design variable density lattice fills to maximize the first eigenfrequency. The effective elasticity of the lattice is used in this process to improve efficiency. Nazir et al. [45] revealed the buckling and post-buckling behavior of different variable-density lattice structures. Takezawa et al. [46] optimized the lattice density distribution to reduce thermal distortion caused by the fabrication process of metal additive manufacturing. The variable density lattice structure plays a great role in regulating the dynamic [47,48,49] and thermodynamic [50,51] properties of the structure. In the design of VDPLMs, there are two key issues to be addressed: the optimal distribution of lattice performance and the relationship between lattice performance and geometrical features, where the latter is particularly critical.

Due to the complex microstructures, it is difficult to obtain the performance of the overall lattice metastructure through analytical solutions. If classical numerical methods are used, a large number of fine grids are required for finite element calculations. Therefore, the multi-scale homogenization method [52,53,54] can be used to obtain the effective mechanical performance of the lattice structure to simplify the calculation. The basic idea of the homogenization method is to treat the non-uniform material as equivalent to an effective homogeneous material, which has the same macroscopic response as the original structure. This homogeneous material can be used to simplify the analysis and calculation in macroscopic analysis. Since the mid-20th century, many scholars have studied homogenization methods. The rule of mixture (ROM) [55] is widely used to predict the material properties and mechanical properties of fiber- or particle-reinforced composites, which can be used as a simple model for rapid prediction by considering only the effect of the relative density without taking into account the specific geometrical features of the lattice. The Voigt–Reuss model [56] provides upper and lower bounds on the effective properties of composites with defined components. The self-consistent method [57,58] sets the volume average properties of inclusions as the values of the macroscopic field to establish a self-consistent assumption, thereby obtaining its effective properties. The Mori–Tanaka method [59,60] establishes the connection between the average strain of the inclusion phase and the average strain of the matrix phase based on the Eshelby equivalent inclusion principle. The above methods have good analytical results when the inclusion content is low and the microstructure complexity is low, but the analytical accuracy makes it difficult to meet the requirements when the microstructure is complex. In this case, the computational homogenization method at the mesoscale [61,62] has better practicability and can accurately calculate the equivalent properties of representative volume elements (RVEs) with complex microstructure. The asymptotic homogenization method [63] is one of the more commonly used methods for numerically solving complex multi-scale problems in the microstructure. This method expands displacement and stress fields asymptotically. It provides effective material properties along with local stress and strain distributions. However, this method is only applicable to relatively simple microstructures. The method of cell (MOC) [64] further divides the representative unit into simple sub-cells to simplify the solution. The MOC substantially improves computational efficiency with a minimal loss in local field accuracy. The Voronoi cell finite element method (VCFEM) [65,66] introduces the Voronoi grid when processing RVEs, which greatly reduces the number of grids compared to traditional grids. The mainstream homogenization methods and their advantages and limitations are summarized in Table 1. Calculations using RVEs require the scale separation assumption [67], meaning the size of the RVE must be much smaller than the macroscopic structure. Additionally, periodic boundary conditions must be applied in all three directions to ensure the RVE is representative. However, in practice, the size of variable density lattice structures is similar to the macroscopic structure, making it impossible to satisfy the scale separation assumption. As a result, the classical homogenization method cannot accurately predict their effective mechanical properties incorporating the surface effect [68,69,70].

Many scholars have studied the effective performance incorporating the surface effect. Le [71] derived an asymptotically exact first-order shear deformation theory for functionally gradient elastic plates of finite thickness using a variational-asymptotic method, taking into account equivalent performance at different thicknesses. This method does not require full periodicity assumptions in three directions. However, to obtain more accurate results, it is often necessary to consider higher-order expansion terms, which results in a high computational complexity. The non-local continuous field theory [72,73,74], which considers a ‘long-range force’ interacting between one point and all points inside the object, has been widely used to analyze the size-dependent mechanical behavior of structures [75,76,77]. Since the surface effect is caused by the free surface in the thickness direction, many scholars have conducted research from the perspective of surface influence. Gurtin et al. [78] developed a mathematical framework for studying the mechanical behavior of material surfaces, considering the surface of nanoporous materials as a zero-thickness film that can withstand tensile forces, and constructed a surface elastic model that takes the surface tensile stiffness into account. Lu et al. [79] proposed a general thin plate theory that includes surface effects, which can be applied to the size-dependent static and dynamic analysis of thin film structures. Wang et al. [80] analyzed the scaling laws of nanostructured materials, which can be used to describe the stiffness hardening and stiffness softening surface effects. As mentioned above, strong surface elasticity has historically been limited to nanoscale materials owing to their high surface-to-volume ratio. Recently, some works [9,81] demonstrated that thin-shell mechanical metamaterials exhibit pronounced surface elasticity even at macroscopic scales. Different from nanoscale surface effects arising from high surface-to-volume ratios, the macro-scale surface effect in lattice metastructures originates from interactions caused by altered force transfer pathways. These alterations stem from the unique microscopic features of the lattice architecture, and the phenomenon is quantified through an intrinsic surface region length scale.

Li and Li [9] investigated a finite-thickness porous lattice plate with a pair of free surfaces at the macroscopic scale and developed an efficient and accurate surface-based multiscale homogenization method to effectively predict the equivalent performance of the lattice structure plate with finite thickness. However, both the nonlocal theory and the surface effect theory describe the size-dependent behavior by introducing a constant parameter related to the microstructure but ignore the surface effect of the variable-density lattice structure changes as the microstructure changes. There is still a gap in applying surface effects to predict the properties of variable-density polymer lattice structures. In VDPLMs, the lattice feature size becomes close to the external feature size, and the surface effect becomes significant. This means that the polymer lattice will exhibit a clear size-dependent behavior. When calculating the effective elastic modulus, it is crucial to account for the influence of the surface elastic layer, which depends on both the lattice feature size and the external feature size. Using the classical homogenization model to predict the effective elasticity in such cases can result in significant errors. Furthermore, since the lattice dimensions and relative density change along the variable-density direction, the properties of the lattice affect the elastic surface thickness, which existing surface elastic models cannot describe. Therefore, to accurately determine the effective properties of variable-density lattice structures, a new surface elastic model that accounts for the density-dependent mechanical behavior is needed.

In this work, we discover that the intrinsic length of surface effects follows a configuration-dependent variable-density surface law in macroscale polymer lattices, demonstrated through high-fidelity finite element simulations. Building on this foundation, we develop a surface-enabled computational homogenization method (SCHM), enabling accurate prediction of macroscopic mechanical behavior in variable-density polymer architectures. This model can be used to analyze the size-dependent and density-dependent mechanical behavior of variable-density lattice metastructures by the computational homogenization method [82,83]. Section 2 states the key problems in formulating a surface elastic model for variable-density lattice metastructures, drawing on the homogenization method of mesoscale mechanics and surface effect theory. Section 3 presents the development of the surface elastic model based on the variable-density surface law of surface intrinsic lengths and proposes a method to calibrate the parameters of the model. Section 4 further demonstrates that the model parameters follow the variable-density surface law and validate the accuracy of the model through its application to a VDPLM. Finally, Section 5 provides the conclusions of the study.

## 2. Problem Statement

### 2.1. Importance of the Variable-Density Surface Effect

**Figure 1:**
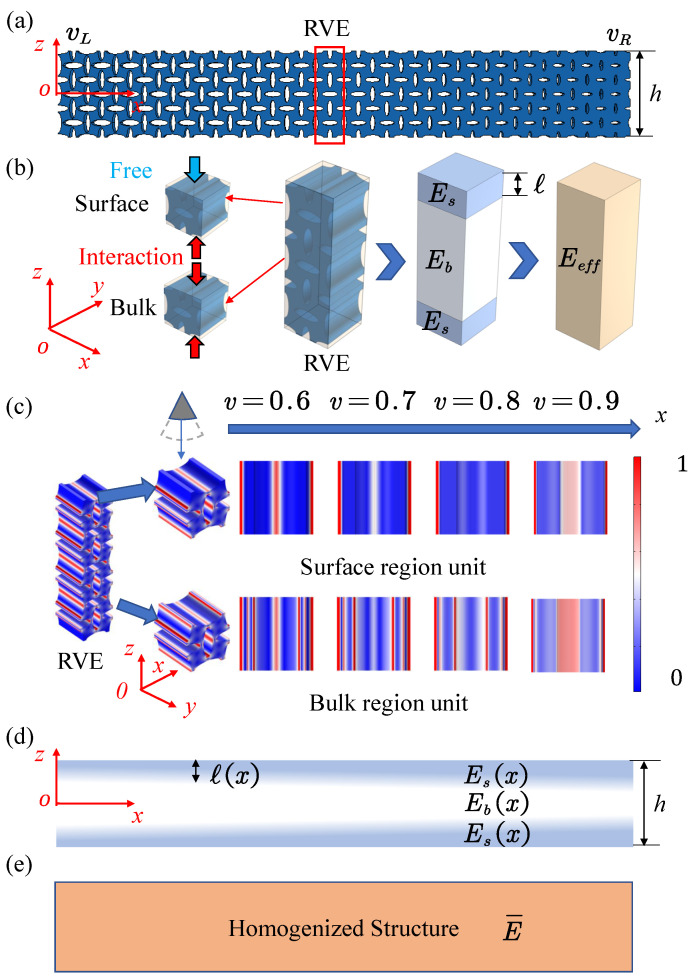
Establishment of a variable-density surface elastic model for VDPLM. (**a**) A VDPLM structure. (**b**) The surface-enabled homogenization method. (**c**) Normalized stress distribution of the surface and bulk region. (**d**) Schematic diagram of the variable-density surface elastic model. (**e**) The homogenized structure.

In this study, an unevenly distributed lattice metastructure is investigated, as shown in Figure 1a. The structure has finite thickness in the *z*-direction and is approximately infinite in the *x*- and *y*-directions. Compared with the bulk lattices, the lattices in the surface region lack the interaction of adjacent lattices. Therefore, the effective elastic modulus of lattices in the surface region is different from that in the bulk region, as shown in Figure 1b. Force conduction paths of VDPLMs with complex microstructures are different from those of homogeneous structures. The elasticity $E_{b}$, $E_{s}$ of the two regions and the thickness *ℓ* of the surface region are jointly influenced by material properties and lattice geometry. Our study focuses on the difference between the bulk modulus and surface modulus, which is mainly governed by the lattice geometry, including lattice type, lattice constant, and relative density. The effective elasticity of the lattice metastructure is determined by both the bulk and surface lattices, with the relative contributions of each being influenced by the ratio of surface thickness *ℓ* to structure thickness *h*.

VDPLMs offer greater flexibility, with their lattice geometry (including lattice constant and relative density) varying along a specific direction. For the VDPLM under axial tension shown in Figure 1a, the normalized stress distribution at different *x*-positions and in different regions is illustrated in Figure 1c. By comparing the lattice stress distributions in the surface and bulk regions at the same x-position, it is evident that the stress distribution near the free surface in the surface region differs from that in the bulk region, showing an obvious surface effect. For the VDPLM, different *x*-positions correspond to different lattice relative densities *v*. With the increase in *v*, the stress distributions in the surface and bulk regions tend to converge. This demonstrates that the lattice geometry distribution significantly influences both the surface elasticity and the surface thickness, as shown in Figure 1d. Therefore, this study specifically investigates the law of equivalent properties as influenced by lattice geometry distribution and structure thickness.

When computing lattice metastructures using the finite element method (FEM), these structures require a highly refined mesh, leading to a significant increase in the degrees of freedom and extended computation times. The computational homogenization method based on the representative volume element (RVE) can be employed to obtain the effective properties of the lattice, addressing the issue of excessive degrees of freedom, but this method relies on the scale separation assumption. For the finite-thickness lattice metastructure bar examined in this paper, the structural size in the thickness direction is comparable to the lattice constant, which violates the scale separation assumption. In practical applications, the variable-density direction is typically perpendicular to the thickness direction of the lattice structure. In the variable-density direction, the structural size is much larger than the lattice constant, thereby satisfying the scale separation assumption. Thus, along the axial direction, the lattice structure is divided into slices of a certain thickness, each containing free boundaries and acting as an RVE. Within each RVE, the lattice metastructure is assumed to be uniformly distributed, effectively simplifying the problem to that of a uniform lattice. The homogenized elasticity of each RVE depends on the lattice geometry and the thickness of the structure. The critical challenge lies in determining the RVE effective modulus for arbitrary cases, which can be derived through the FEM-based homogenization method for discrete cases. Then, the continuous effective modulus can be established through methods such as induction, fitting, or machine learning [84,85]. Since the loading direction aligns with the variable-density direction, the axial stresses in each homogenized slice are equal in magnitude, and the total stress σ satisfies

(1) $$\sigma =\sigma_{i}\;\mathrm{for}i=1,2,\ldots ,N,$$

where $\sigma_{i}$ is the stress on homogeneous slice *i*, and the total strain ε is the sum of the individual slice strains:

(2) $$\varepsilon =\frac{1}{L}\sum_{i=1}^{N}\varepsilon_{i}\Delta x,$$

where $\varepsilon_{i}$ is the strain of slice *i*, Δx is the thickness of the slice, and *L* is the axial characteristic length of the lattice metastructure. The elastic modulus $\bar{E}$ of the structure shown in Figure 1d can be obtained by substituting the intrinsic relationship as follows:

(3) $$\bar{E}=\frac{1}{\frac{1}{L}\sum_{i=1}^{N}\frac{1}{E_{eff}^{i}}\Delta x},$$

where $E_{eff}^{i}$ is the effective elastic modulus of slice *i*. The continuous form can also be used as

(4) $$\bar{E}=\frac{1}{\frac{1}{L}\int_{L}\frac{1}{E_{eff}\left(x\right)}dx},$$

where $E_{eff}\left(x\right)$ is the effective elastic modulus of the slice at position *x*.

### 2.2. Key Problems and Contributions

In summary, this study focuses on solving the following three problems to efficiently and accurately predict the effective mechanical properties of VDPLMs.

(1) VDPLMs exhibit an obvious surface effect when the lattice constant and the macroscopic size of the metastructure are of the same order of magnitude. However, the relationship between the microstructure and surface thickness is unclear, limiting their design and application. Therefore, at the scientific level, this paper aims to uncover the variable-density surface law governing the surface thickness of polymer metastructures with variable density.
(2) Existing surface models that assume a constant surface thickness cannot accurately describe their size-dependent mechanical behavior. Therefore, at the theoretical level, it is necessary to establish a variable-density surface elastic model to describe the mechanical behavior of variable-density microstructures.
(3) VDPLMs have complex microstructures, and using the high-fidelity FEM to fully resolve these microstructures requires substantial computational resources and time. Therefore, to predict the mechanical properties of polymer metastructures incorporating surface effect due to the surface effect with both high efficiency and accuracy, at the technical level, it is essential to develop a homogenization method that accounts for the variable-density surface effect.

This work advances the understanding and predictive modeling of variable-density polymer lattice metastructures through two key contributions:

(a) Scientific breakthrough: Current limitations in predicting mechanical performance stem from incomplete knowledge of surface-induced size effects of variable-density polymer lattice metastructures. By employing high-fidelity finite element simulations, we reveal a novel variable-density surface law governing the surface intrinsic length at macroscopic scales, which is fundamentally determined by the lattice configuration.
(b) Methodological innovation: Traditional homogenization methods based on scale separation prove inadequate when macroscopic dimensions approach surface intrinsic lengths. To overcome this, we develop a surface-enabled computational homogenization framework informed by our variable-density surface law discovery. This new approach enables efficient macroscopic prediction of mechanical behaviors while preserving critical configuration-dependent surface effects.

**Figure 2:**
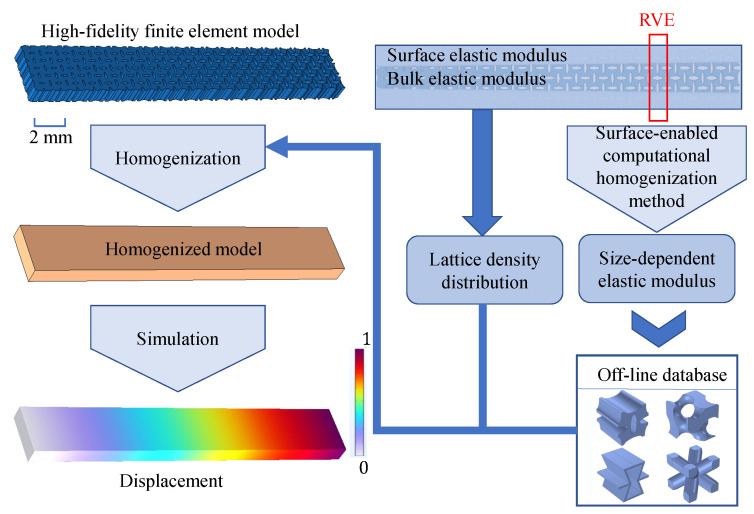
Technical route of the construction of the variable-density surface elastic model for VDPLM incorporating the surface effect.

The above problems can be addressed by constructing a variable-density surface elastic model based on an offline database, as the framework shown in Figure 2. First, an offline database is created, consisting of numerous microstructures along with their effective elastic modulus and bulk elastic modulus, generated through high-throughput numerical computations. Next, the variable-density surface law obeyed by the surface intrinsic length is analyzed according to the offline dataset. Subsequently, an effective mechanical model for variable-density polymer metastructures, which incorporates surface effects, is developed by applying a homogenization method in conjunction with the variable-density surface law derived from the obtained surface intrinsic lengths. Finally, using the offline database and the effective mechanical model, the effective elasticity of VDPLM can be accurately and efficiently predicted online, offering valuable insights for design optimization.

Lattice metastructures, such as aircraft lattice wings and satellite lattice boxes, exhibit microstructure-dependent mechanical behavior. However, high-fidelity finite element analysis is computationally expensive and impractical. By homogenizing RVE, an offline database of effective elastic modulus is constructed, enabling the derivation of the variable density law for RVE. This law facilitates the development of a surface elastic model for lattice metastructures, enabling both mechanical behavior prediction and inverse design.

## 3. A Homogenization Method for the VDPLM Incorporating the Surface Effect

**Figure 3:**
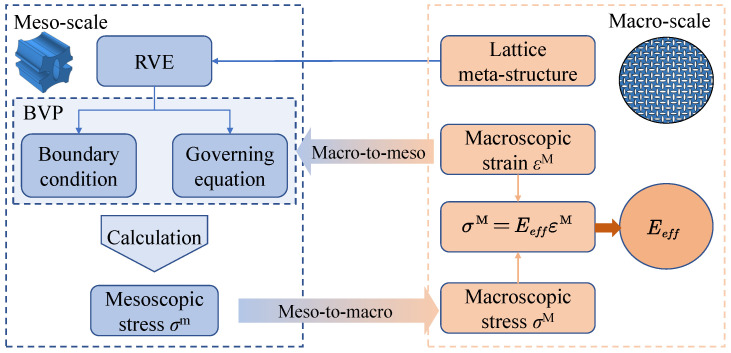
Flow chart of the homogenization process.

In this section, a homogenization method for VDPLM under stretching, incorporating surface effect, is developed. First, based on the revealed variable-density surface law governing the surface thickness, we establish a variable-density surface elastic model to describe the size-dependent and density-dependent behavior of VDPLM. Next, by considering macroscopic deformation, we define the boundary value problem of the RVE and establish the transformation from macroscopic to mesoscopic. The boundary value problem is solved using the finite element method, and then the transformation from the mesoscale to the macroscale is established based on the Hill–Mandel condition to obtain the effective performance of the RVE. The homogenization process is shown in Figure 3. In the following description, the superscripts m and M represent the mesoscale and macroscale quantities, respectively.

### 3.1. A Variable-Density Surface Law

When the lattice constant is much smaller than the external feature size, the lattice properties approximate those of the bulk region. However, as the lattice size approaches the external feature size, the failure mode changes, with the influence of the surface region increasing. This behavior applies to many physical properties. The ratio of the physical properties of the lattice metastructure (denoted by $F\left(h\right)$) to those of the bulk region (denoted by $F_{b}$) at small external feature sizes *h* can be expressed in terms of a dimensionless variable $X_{j}\left(j=1,\ldots M\right)$ and a size-dependent dimensionless parameter $k_{in}/h$.

(5) $$\frac{F\left(h\right)}{F_{b}}=F\left(X_{j},k_{in}/h\right),$$

where $k_{in}$ is a surface intrinsic length related to the surface properties, *h* is the external feature size of the structure under consideration, and $F_{b}$ is the bulk lattice property when the lattice size is much smaller than the external feature size, where the influence of the surface layer can be neglected. The expansion of $F(X_{j},k_{in}⁄h)$ is provided as follows:

(6) $$\frac{F\left(h\right)}{F_{b}}=1+\alpha \frac{k_{in}}{h}+O{\left(\frac{k_{in}}{h}\right)}^{2},$$

where $O{\left(k_{in}/h\right)}^{2}$ denotes the second-order remainder, which can be neglected when $k_{in}⁄h\ll 1$. The above equation can thus be rewritten as follows:

(7) $$\frac{F\left(h\right)}{F_{b}}=1+\alpha \frac{k_{in}}{h}.$$

In this study, the property of interest is the elasticity modulus, so *F* is replaced with *E* in the above equation as follows:

(8) $$\frac{E\left(h\right)}{E_{b}}=1+\alpha \frac{k_{in}}{h},$$

where E(h) is the effective elastic modulus of the uniform density lattice metastructure with thickness *h*, and $E_{b}$ is the bulk lattice effective elastic modulus.

For VDPLM, the lattice relative density changes along the variable density direction. As a result, the surface intrinsic length and bulk elastic modulus are position-dependent within this direction.Therefore, we can obtain the position-dependent effective modulus as follows:

(9) $$\frac{E\left(h,x\right)}{E_{b}\left(x\right)}=1+\alpha \frac{k_{in}\left(x\right)}{h}.$$

E(h) and $E_{b}$ have different values for different lattice geometries and materials. However, the relationship between them still satisfies Equation (9). Therefore, we calculate the values of E(h) and $E_{b}$ of different lattice geometries and store them as an offline database. In the database, the lattice geometry is part of the input. The relationship between lattice geometry and effective elastic modulus is established by fitting or machine learning, and then the $k_{in}$ under different geometries is obtained. Therefore, we can analyze the variable-density surface law of different lattice geometries.

### 3.2. Effective Elastic Modulus Incorporating the Surface Effect of the VDPLM

In Section 2, we observe that both lattice geometry and lattice position influence the stress distribution of the lattice, and for variable-density lattice structures, this effect varies with the coordinates. In this section, the variable-density surface elasticity model is employed to analyze the elastic modulus of the surface and bulk regions. Additionally, the relationship between the effective elastic modulus of the lattice structure and the density distribution, as well as the thickness *h* of the structure, is established.

The lattice metastructure consists of a bulk region and a surface region. In the Cartesian coordinate system, the *z*-direction represents the thickness direction, while the *x*-direction corresponds to the variable density direction. $l\left(x\right)$ denotes the surface thickness at position *x*, which is influenced by a combination of the lattice type, the lattice constant, and the relative density of the lattice.

The lattice geometry of a VDPLM varies along the axial direction, meaning that the lattice constant *a* and the relative density *v* change with respect to the coordinate *x*. In the VDPLM, both *a* and *v* are functions of the coordinate *x*, i.e.,

(10) $$\begin{array}{c} v=v\left(x\right),\\ a=a\left(x\right). \end{array}$$

Therefore, the following simplification can be made:

(11) $$\begin{array}{c} E_{b}\left(a,v\right)\to E_{b}\left(x\right),\\ E_{s}\left(a,v\right)\to E_{s}\left(x\right),\\ E_{eff}\left(a,v\right)\to E_{eff}\left(x\right), \end{array}$$

where $E_{b}\left(x\right)$ is the effective elastic modulus of the bulk region lattice, $E_{s}\left(x\right)$ is the effective elastic modulus of the surface region lattice, and $E_{eff}\left(x\right)$ is the effective elastic modulus of the VDPLM. Therefore, the elasticity of the two regions is also position-dependent and can be expressed by the following equation:

(12) $$E_{eff}\left(x\right)=\left\{\begin{array}{c} E_{b}\left(x\right),\;\left|z\right|<h/2-l\left(x\right)\\ E_{s}\left(x\right),\;h/2-l\left(x\right)<\left|z\right|<h/2 \end{array}\right.,$$

where *h* is the lattice metastructure thickness, and $l\left(x\right)$ is the surface thickness. According to the assumption of elastic modulus, the effective elastic modulus of the lattice metastructure is determined by both the surface region and the bulk region.

### 3.3. Surface-Enabled Computational Homogenization Method

The density distribution of the lattice metastructure causes the elasticity of the surface region to vary along the axial direction, making it difficult to calculate the effective elasticity using the classical surface elastic model.

#### 3.3.1. Macro-to-Meso Scale Transition

In the classical multiscale theoretical framework, the displacement *u* at the mesoscopic scale is determined by the average strain $\bar{\varepsilon }$ and the disturbance displacement $u^{*}$ caused by the mesoscopic deformation. The displacement can be assumed to be

(13) $$u\left(x\right)=\bar{\varepsilon }\left(x-x_{0}\right)+u^{*}\left(x\right),$$

where $\bar{\varepsilon }\left(x-x_{0}\right)$ denotes the linear displacement due to the mean strain, $u^{*}\left(x\right)$ denotes the disturbance displacement by other adjacent lattices due to the heterogeneity of the metastructure, $x_{0}$ denotes the reference point position, and *x* denotes the position of the point of interest. Each of the following physical quantities in this subsection represents the *x*-direction component by default.

The RVE boundary strain is the key to the macro-to-meso scale transition, and taking the derivative of Equation (13) concerning the *x*, we can obtain

(14) $$\varepsilon \left(x\right)=\bar{\varepsilon }+\varepsilon^{*}\left(x\right).$$

Taking the volume average of mesoscopic quantities over RVEs, we can obtain

(15) $$\frac{1}{V}\int_{x\in V}\varepsilon \left(x\right)dx=\bar{\varepsilon }+\frac{1}{V}\int_{x\in V}\varepsilon^{*}\left(x\right)dx,$$

where *V* is the volume of the RVE, and Equation (15) can be simplified by treating the integral term on the right-hand side of the equation according to the divergence theorem as

(16) $$\frac{1}{V}\int_{x\in V}\varepsilon \left(x\right)dx=\bar{\varepsilon }+\frac{1}{V}\int_{x\in S}u^{*}\left(x\right)dx,$$

where *S* is a pair of parallel boundaries in the *x*-direction, and usually

(17) $$\bar{\varepsilon }:=\frac{1}{V}\int_{x\in V}\varepsilon \left(x\right)dx.$$

Combined with Equation (16), the macro-to-meso scale transition constraint condition can be obtained:

(18) $$\int_{x\in S}u^{*}\left(x\right)dx=0.$$

Therefore, periodic boundary conditions (PBCs) can be applied in the *x*- and *y*-directions according to the macro-to-meso scale transition constraint and free boundary in the *z*-direction. The disturbance displacements on the parallel pairs of boundaries can be written as follows:

(19) $$\begin{array}{c} u^{*}\left(x=0\right)=u^{*}\left(x=a\right),\\ u^{*}\left(y=0\right)=u^{*}\left(y=a\right). \end{array}$$

From Equation (19), the displacement difference between the boundary pairs in the *x* and *y* directions can be written as follows:

(20) $$\begin{array}{c} u_{x}\left(x=a\right)-u_{x}\left(x=0\right)=a\frac{\partial u_{x}}{\partial x},\\ u_{y}\left(y=a\right)-u_{y}\left(y=0\right)=a\frac{\partial u_{y}}{\partial y}, \end{array}$$

where the subscripts *x* and *y* represent the *x*- and *y*-directions, and the above equation describes the displacement boundary conditions for the RVE. In addition to the displacement boundary conditions, these parallel boundary pairs should also meet the traction boundary conditions:

(21) $$\begin{array}{c} \sigma_{x}\left(x=0\right)+\sigma_{x}\left(x=a\right)=0,\\ \sigma_{y}\left(x=0\right)+\sigma_{y}\left(y=a\right)=0. \end{array}$$

The equilibrium equations can be written as

(22) $$\nabla \cdot \sigma^{m}=0.$$

The zero-traction boundary conditions are applied at the free surfaces of the RVE according to the macroscopic boundary conditions, i.e.,

(23) $$\sigma_{z}\left(z=0\right)+\sigma_{z}\left(z=a\right)=0.$$

#### 3.3.2. Meso-to-Macro Scale Transition Using the Hill–Mandel Condition

The stress field inside the lattice is typically complex, and the primary interest often lies in the average properties of the lattice, which is the main focus of homogenization. Under the small deformation assumption, the constitutive equation for linear elastic materials can be written as follows:

(24) $$\sigma^{m}=E\varepsilon^{m},$$

where the mesoscopic strain $\varepsilon^{m}$ is defined as

(25) $$\varepsilon^{m}=\nabla_{x}u^{m},$$

where $u^{m}$ represents the mesoscopic displacement. The condition (18), which is satisfied by the boundary disturbance displacements, ensures the consistency of the meso-to-macro scale transition. This corresponds to the Hill–Mandel condition [86], which is defined as follows:

(26) $$\sigma^{M}\varepsilon^{M}=\frac{1}{V}\int_{V}\sigma^{m}\varepsilon^{m}dV.$$

To calculate the effective elastic modulus of the RVE, an effective strain $\varepsilon^{M}$ (macroscopic strain) is applied to the boundary pair along the *x*-direction of the RVE.

(27) $$u\left(x=a\right)-u\left(x=0\right)=a\varepsilon^{M},$$

where u(x=a) and u(x=0) represent the displacements at a pair of parallel boundaries in the *x*-direction of the lattice, and *a* is the lattice constant. With macroscopic strain, the macroscopic effective stress can be obtained by volume average of microscopic stress [87]:

(28) $$\sigma^{M}=\frac{1}{V}\int_{V}\sigma^{m}dV.$$

After obtaining the macroscopic strain and macroscopic effective stress, the effective elastic modulus of the RVE can be determined using the following homogenized constitutive equation:

(29) $$\sigma^{M}=E_{eff}\varepsilon^{M},$$

where $E_{eff}$ is the effective modulus. For a VDPLM, the effective modulus is position-dependent and is denoted by $E_{eff}\left(x\right)$.

### 3.4. Calibration Method for the Surface Intrinsic Length

#### 3.4.1. Effective Elastic Modulus

**Figure 4:**
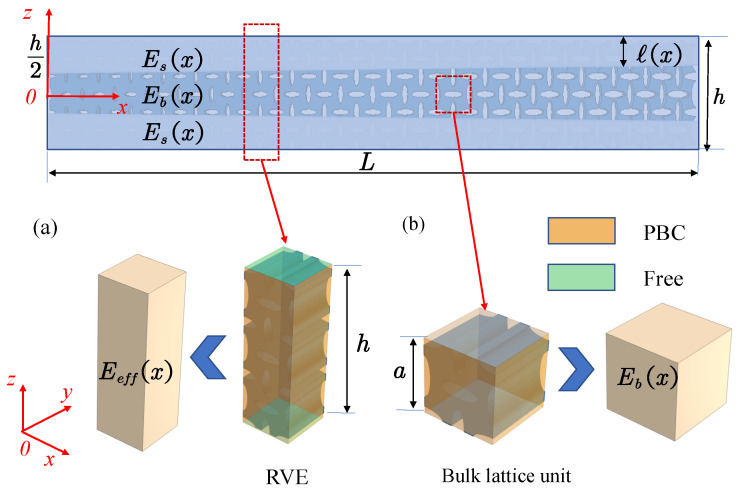
Boundary value problems. (**a**) Boundary value problem of the RVE. (**b**) Boundary value problem of the bulk lattice unit. A zero-traction boundary condition is applied on the free surfaces.

Consider the homogeneous slice RVE shown in Figure 4 as the object of analysis. The effective strain $\varepsilon^{M}$ (macroscopic strain) is applied to the *x*-direction boundary pairs. The RVE is divided into the surface and bulk regions. The macroscopic average stress $\bar{\sigma }$ consists of two components: surface stress $\sigma_{s}$ and body stress $\sigma_{b}$.

(30) $$\bar{\sigma }=\frac{1}{V_{s}+V_{b}}\left(\int_{V_{s}}\sigma_{s}dV_{s}+\int_{V_{b}}\sigma_{b}dV_{b}\right),$$

where $V_{s}$ is the volume of the surface area, $V_{b}$ is the volume of the bulk area, and $\sigma_{s}$ and $\sigma_{b}$ are the macroscopic local stresses of the surface area and the bulk area after homogenization, respectively, and are defined by the following formula:

(31) $$\begin{array}{c} \sigma_{s}=\frac{1}{V_{s}}\int_{V_{s}}\sigma^{m}dV_{s},\\ \sigma_{b}=\frac{1}{V_{b}}\int_{V_{b}}\sigma^{m}dV_{b}. \end{array}$$

The constitutive equation at the macroscopic scale is provided by the following equation:

(32) $$\begin{array}{c} \sigma_{s}=E_{s}\varepsilon_{s},\\ \sigma_{b}=E_{b}\varepsilon_{b}, \end{array}$$

where $E_{s}$ and $E_{b}$ denote the surface elastic modulus and the bulk elastic modulus, respectively, and the effective elastic modulus incorporating the surface effect can be obtained by combining Equations (29) and (30). Combining the surface elastic model and Equations (30) and (32), the effective elastic modulus of the RVE can be written in the following form:

(33) $$E_{eff}\left(x\right)=v_{s}\left(x\right)E_{s}\left(x\right)+v_{b}\left(x\right)E_{b}\left(x\right),$$

where $v_{s}\left(x\right)$ and $v_{b}\left(x\right)$ are the surface region volume fraction and the bulk region volume fraction at position *x*. The effective elastic modulus can be obtained from the following equation:

(34) $$E_{eff}\left(x\right)=\frac{2l\left(x\right)}{h}E_{s}\left(x\right)+\left(1-\frac{2l\left(x\right)}{h}\right)E_{b}\left(x\right),$$

where combining Equations (8) and (34) provides the following equation:

(35) $$\frac{E_{eff}\left(x,h\right)}{E_{b}\left(x\right)}=1+2\frac{k_{in}\left(x\right)}{h},$$

where

(36) $$k_{in}\left(x\right)=\left(\frac{E_{s}\left(x\right)}{E_{b}\left(x\right)}-1\right)l\left(x\right).$$

It should be noted that $\left(E_{s}\left(x\right)⁄E_{b}\left(x\right)-1\right)$ can be either positive or negative, leading to corresponding positive or negative values of $k_{in}\left(x\right)$, depending on the relative value of $E_{s}\left(x\right)$ and $E_{b}\left(x\right)$. When $k_{in}\left(x\right)>0$, the surface effect strengthens the effective elastic modulus $E_{eff}\left(x\right)$, while the opposite is true when $k_{in}\left(x\right)<0$, which weakens the elastic modulus. The surface intrinsic length $k_{in}\left(x\right)$ is determined by the lattice geometry. The ratio of the surface intrinsic length to the external feature size $k_{in}\left(x\right)⁄h$ dictates the strength of the surface effect. When $|k_{in}⁄h|\ll 1$, $E_{eff}\left(x\right)/E_{b}\left(x\right)\to 1$. Conversely, when $|k_{in}⁄h|\approx 1$, the surface effect becomes more obvious.

#### 3.4.2. Implementation of the FEM-Based Homogenization Method

This subsection describes the procedure for solving the bulk and size-dependent elastic modulus of lattices using the FEM. An FEM-based homogenization method is proposed to calibrate the surface intrinsic lengths.

To meet the scale separation assumption, the PBC is applied to the boundary pairs in these directions. This boundary condition allows for the simulation of the actual lattice behavior in the bulk region. The average stress in the bulk is obtained by applying a unit strain in the x-direction to the RVE, and the bulk elastic modulus can be obtained.

When calculating the effective elastic modulus $E_{eff}$ for lattice slices with different thicknesses, a free boundary is applied in the thickness direction (*z*-direction), while the PBC is applied in the *x*- and *y*-directions to satisfy the scale-separation assumption in the multiscale analysis method. In this case, the homogenization method incorporating the surface effect is more accurate than the classical homogenization method. A certain strain is applied to the RVE, and the average stress is obtained through finite element calculations. The effective elastic modulus $E_{eff}$, considering the surface effect, is then determined.

**Figure 5:**
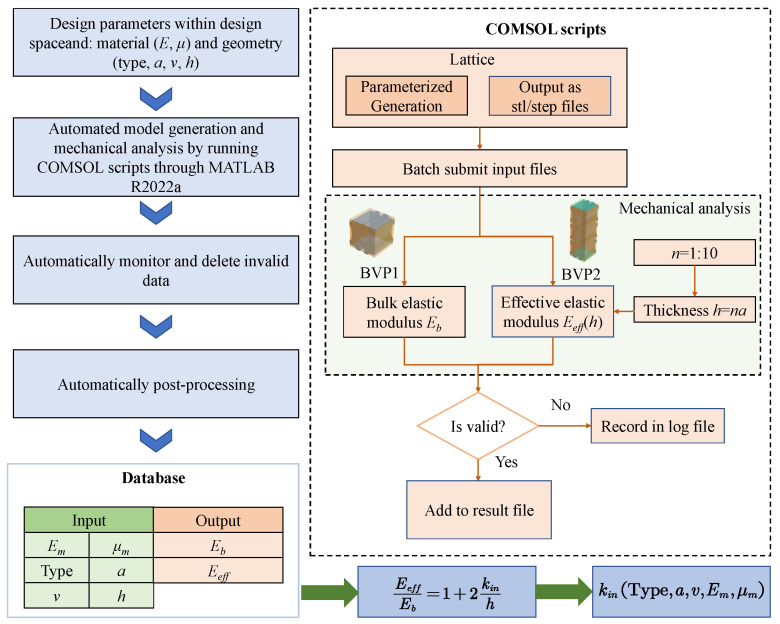
Construction process of an offline database.

**Figure 6:**
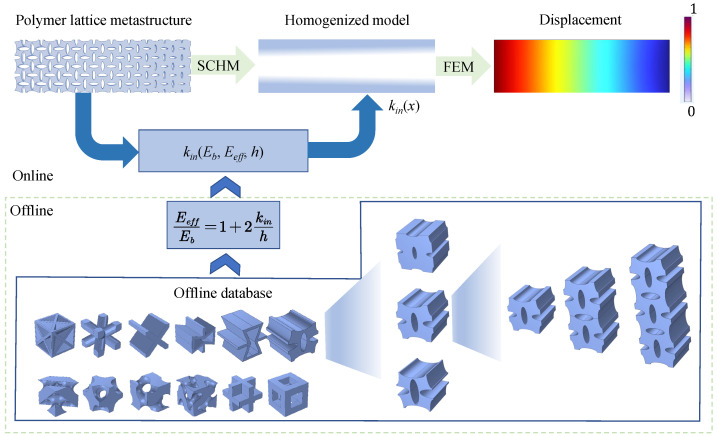
Construction and use process of the surface intrinsic length.

The database of the bulk elastic modulus $E_{b}$ and effective elastic modulus $E_{eff}$ at different design parameters can be obtained using the method described above. The offline database construction process is shown in Figure 5. Equation (35) can then be fitted to determine the surface intrinsic length $k_{in}$. For the VDPLM, both the relative density and lattice constant vary. Therefore, it is necessary to calculate and generate a database of $E_{b}$ and $E_{eff}$ for different relative densities and lattice constants, which can then be fitted to obtain $k_{in}$, as shown in Figure 6.

The distributions of the relative density *v* and lattice constant *a* in the VDPLM are coordinate-dependent, i.e., a=a(x) and v=v(x). Using the fitted functions $k_{in}\left(a,v\right)$ and $E_{b}\left(a,v\right)$, along with the lattice metastructure thickness *h*, and combining with Equation (35), the size-dependent elastic modulus $E_{eff}\left(x\right)$ of the RVE at different positions *x* can be obtained.

Finally, for a VDPLM, the structural effective elastic modulus $\bar{E_{eff}}$ can be obtained from Equation (4). This value can then be used to predict the structure’s deformation under different loading.

## 4. Results and Discussion

This section further explores the variable-density surface law followed by the surface intrinsic length through high-throughput simulations. The variable-density surface elastic model is applied to design and predict the size-dependent mechanical behavior of the VDPLM. The materials used in the following studies are vitrimers, characterized by the following physical parameters: $E_{m}=4.8\;\mathrm{GPa}$, μ=0.3, $\rho =1424\;\mathrm{kg}/m^{3}$, and the lattice constant a=1mm.

As a phononic crystal material, the two-dimensional crisscrossed elliptical hole lattice metastructure exhibits both load-bearing and vibration-damping properties, while its simple design makes it easy to fabricate. Therefore, this section focuses on analyzing this lattice and investigating the variation of the effective elastic modulus under different lattice geometry and structure thicknesses.

**Figure 7:**
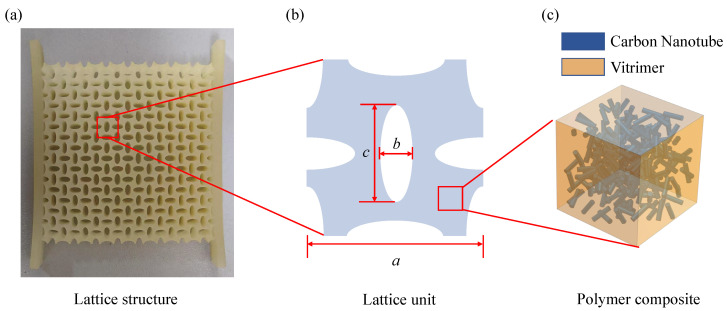
Crisscrossed elliptical holes lattice structure. (**a**) Lattice structure. (**b**) Lattice unit. (**c**) Polymer or polymer-matrix composite material.

The characteristic geometry of the crisscrossed elliptical hole lattice is square and is defined by three parameters: the lattice constant *a*, the minor axis length *b*, and the major axis length *c*, as shown in Figure 7. For simplicity, c=3b is imposed to reduce the number of structural parameters. The relative density *v* is controlled by parameter *b*, while the lattice constant is adjusted using parameter *a*. The mapping relationship between *b* and the relative density *v* can be derived through geometrical calculations, as shown below:

(37) $$v=1-3\pi b^{2}/a^{2}.$$

The input for the parametric generation of the lattice is the relative density *v*, and the output is the structural parameter b/a. Therefore, the above equation needs to be transformed into

(38) $$\frac{b}{a}=\sqrt{\frac{1-v}{3\pi }}.$$

The geometrical features of the lattice structure limit the structural parameters, where b⁄a ranges from 0 to 0.25; therefore, the relative density ranges from 0.41 to 1.

The main factors affecting lattice properties are the material parameters and lattice geometry parameters. In this paper, we focus on static linear elastic deformation, where the material parameters considered are the elastic modulus and Poisson’s ratio. The effects of material elastic modulus, material Poisson’s ratio, lattice constant, and relative density on the effective elastic modulus, bulk elastic modulus, and surface intrinsic length are analyzed separately.

### 4.1. Effect of Material Parameters on the Effective Elastic Modulus

**Figure 8:**
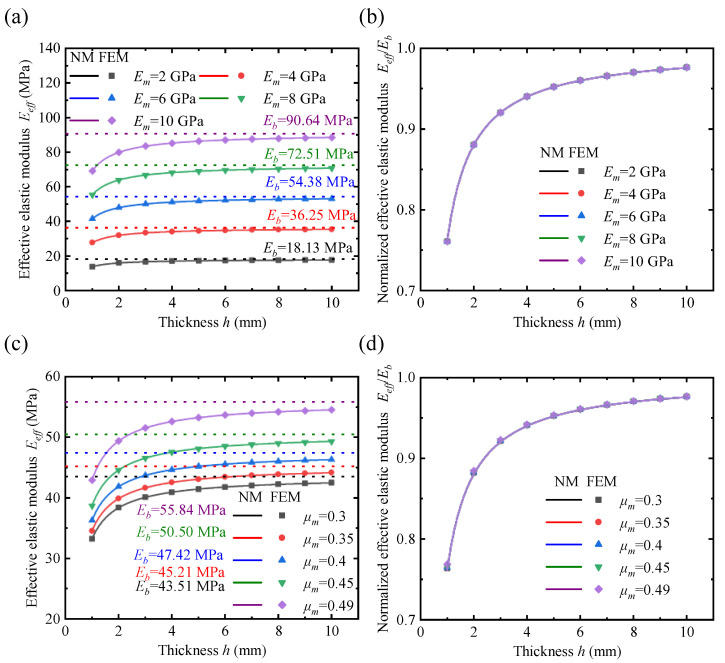
Effect of material properties on the effective elastic modulus and normalized effective elastic modulus. (**a**) Effective elastic modulus with different elastic moduli of materials. (**b**) Normalized effective elastic modulus with different elastic moduli of materials. (**c**) Effective elastic modulus with different Poisson’s ratios of materials. (**d**) Normalized effective elastic modulus with different Poisson’s ratios of materials.

The variation of the effective elastic modulus $E_{eff}$ and the normalized effective elastic modulus $E_{b}/E_{eff}$ for lattice metastructures with different thicknesses *h* is analyzed under different material parameters: the elastic modulus $E_{m}$ and Poisson’s rations $\mu_{m}$ values. In Figure 8a, it can be seen that the lattice exhibits different bulk elastic moduli for varying $E_{m}$ values. As the thickness increases, the effective elastic modulus converges to the bulk elastic modulus value from a smaller initial value. An obvious surface effect is observed. Analysis of several curves for different $E_{m}$ values shows the same trend, though the quantification pattern is not clear. To better analyze the effect of surface elasticity, the effective elastic modulus is normalized, as shown in Figure 8b. The normalized effective elastic modulus curves for different $E_{m}$ values overlap in the figure, indicating that the surface intrinsic length $k_{in}$ in the surface elastic model is constant, i.e., $k_{in}\left(E_{m}\right)=k_{in}^{con}$. Analyzing the effect of Poisson’s ratio of the material on $E_{eff}$ in Figure 8c, we observe that the trend is similar to that of the normalized effective elastic modulus curves in Figure 8a. The bulk elastic modulus changes by about 22% within the typical Poisson’s ratio range from 0.3 to 0.49 for common engineering materials, while the normalized effective elastic modulus curves essentially overlap. Therefore, it can be concluded that material parameters affect the bulk elastic modulus, with $E_{m}$ being the dominant factor. However, these parameters have little influence on the surface intrinsic length $k_{in}$, which characterizes the strength of the surface effect. Based on this, we can obtain the following simplification:

(39) $$k_{in}\left(E_{m},\mu_{m}\right)\to k_{in}^{\mathrm{con}}.$$

### 4.2. Effect of Lattice Geometry on the Effective Elastic Modulus

**Figure 9:**
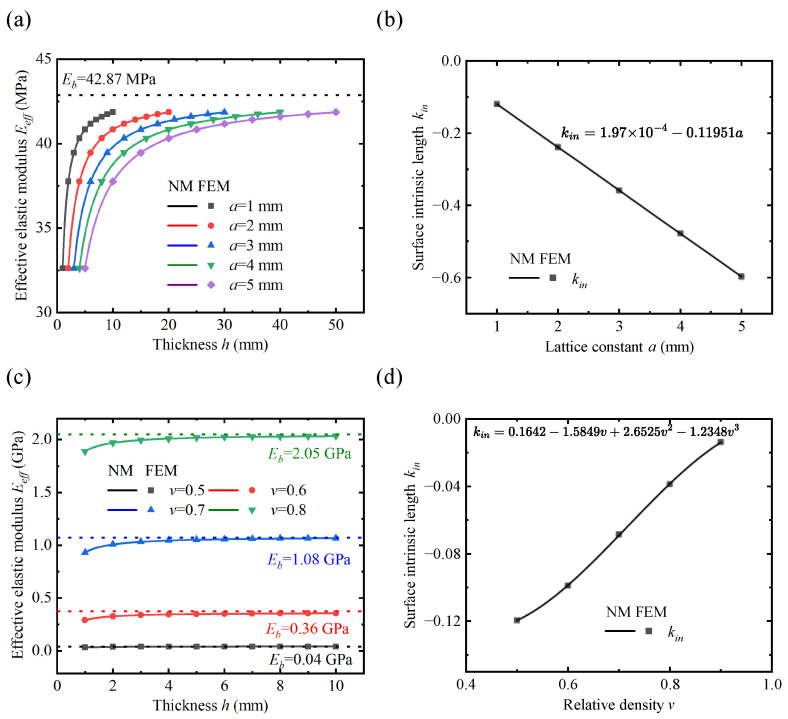
Effect of lattice geometry on the effective elastic modulus and surface intrinsic length. (**a**) Effective elastic modulus with different lattice constants (*v* = 0.5). (**b**) The surface intrinsic length with different lattice constants. (**c**) Effective elastic modulus with different relative densities of lattices (*a* = 1). (**d**) The intrinsic parameter with different relative densities of lattices.

Lattice constant a is one of the important parameters of lattice geometry. Figure 9a shows the $E_{eff}$ curves for different values of *a*. It can be seen that the $E_{eff}$ curves converge to the same value of $E_{b}$ as the thickness increases, indicating that the lattice constant has little effect on the value of $E_{b}$. This is likely because the bulk elastic modulus represents the effective elastic modulus under the PBC, where the effect of the lattice constant on the stress–strain relationship can be neglected. Therefore, $E_{b}\left(v,a\right)$ can be simplified to $E_{b}\left(v\right)$. To quantitatively analyze the strength of the surface effect, the surface intrinsic lengths at different lattice constants were obtained by fitting according to Equation (35), as shown in Figure 9b. Analyzing the influence of the lattice constant on the surface intrinsic length reveals a clear linear relationship. After linear fitting, the ratio of the absolute value of the constant term to the coefficient of the primary term is 0.0016, indicating a positive proportional relationship between the surface intrinsic length and the lattice constant. This is because the lattice size does not affect the stress distribution, and the ratio of the surface thickness *ℓ* to the lattice constant a remains constant, so $k_{in}/a=\mathrm{const}$. Another important parameter of the lattice geometry is relative density *v*. Figure 9c shows the variation of the effective elastic modulus of the lattice metastructure under different relative densities *v*. A more obvious surface effect is observed at smaller thicknesses. As the relative density increases from 0.5 to 0.8, the value of $E_{b}$ increases by approximately 50 times, indicating that *v* is a major factor influencing the bulk elastic modulus. Fitting the results with a third-order polynomial can yield better results, as shown in Figure 9d. In summary, the effect of relative density on the bulk elastic modulus and surface intrinsic length is more complex and requires specific analysis for different lattices. In contrast, the influence of the lattice constant on both the bulk elastic modulus and surface intrinsic length follows a simpler pattern. Therefore, the two model parameters can be decoupled and simplified using the following equation:

(40) $$\begin{array}{c} \left\{\begin{array}{c} E_{b}\left(v,a\right)\to E_{b}\left(v\right)\\ k_{in}\left(v,a\right)\to k_{in}\left(v\right)a \end{array}\right.. \end{array}$$

**Figure 10:**
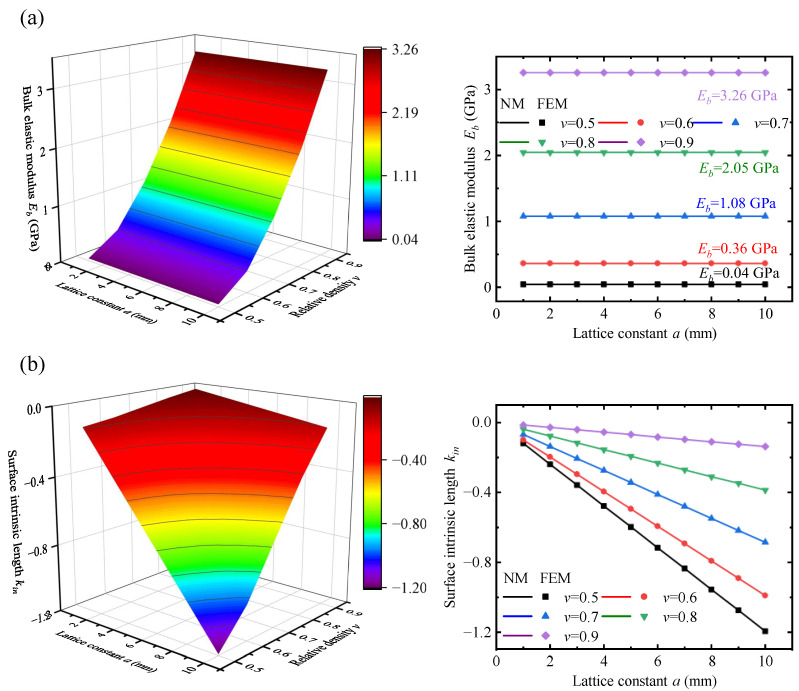
The combined effect of relative density and lattice constant to the bulk elastic modulus and surface intrinsic length. (**a**) Bulk elastic modulus mapping with variable lattice constants and relative densities. (**b**) The surface intrinsic length mapping with variable lattice constants and relative densities.

The above analysis discusses the effects of single-parameter variations in the lattice constant and the surface intrinsic length. Now, the composite effects of both parameters on the bulk elastic modulus and surface intrinsic length are analyzed to verify the simplified law proposed earlier. In Figure 10, it is shown that different values of *v* correspond to different $E_{b}$ values. As *v* approaches 1, $E_{b}$ becomes closer to the material parameter $E_{m}$. This is because as *v* increases towards 1, the pore characteristics decrease geometrically, and the material tends to behave like a homogeneous material. Additionally, $E_{b}\left(a\right)$ remains constant for different *v* values as lattice constant *a* increases. For the surface intrinsic length, $k_{in}^{v}\left(a\right)$ shows a clear positive proportionality at different *v* values. The smaller the value of *v*, the larger the absolute value of the surface intrinsic length. This is because a smaller *v* leads to a change in the surface region failure form, with more energy competition with the bulk region, resulting in a more significant surface effect. In summary, the values of *v* and affect $E_{b}$ and $k_{in}$ independently, which verifies the reliability of the simplified results in Equation (40).

Taken together, the following decoupling simplifications can be made to reduce the amount of computation required for the database calculation process:

(41) $$\left\{\begin{array}{c} E_{b}\left(E_{m},\mu_{m},v,a\right)\to E_{b}\left(E_{m},\mu_{m},v\right)\\ k_{in}\left(E_{m},\mu_{m},v,a\right)\to k\left(v\right)a \end{array}.\right.$$

Therefore, Equation (35) can be written as follows:

(42) $$E_{eff}\left(E_{m},\mu_{m},v,a\right)=\left[1+2\frac{a}{h}k\left(v\right)\right]E_{b}\left(E_{m},\mu_{m},v\right).$$

In practical applications, the materials in the lattice metastructure are mostly made of the same material. When the geometric effect is mainly considered, the above formula can be simplified to the following:

(43) $$E_{eff}\left(v,a\right)=\left[1+2\frac{a}{h}k\left(v\right)\right]E_{b}\left(v\right).$$

**Figure 11:**
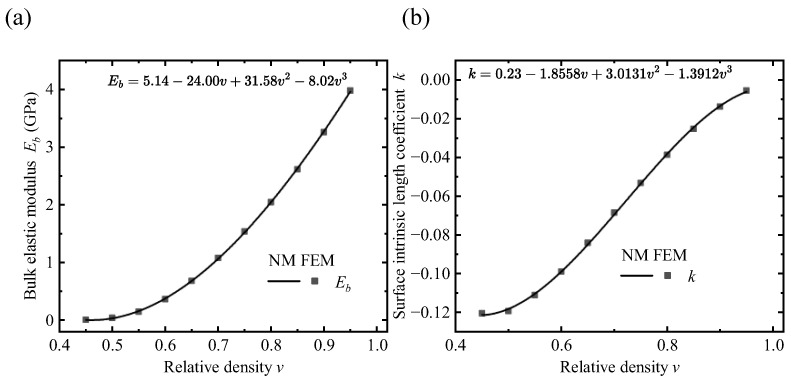
Mapping of the crisscrossed elliptical holes lattice *v* to $E_{b}$, *k*. (**a**) Mapping from *v* to $E_{b}$. (**b**) Mapping from *v* to *k*.

Using the variable-density surface elastic model, we can predict the effective elastic modulus of the VDPLM bar for different parameter combinations (v,a,h) after obtaining the mapping relationships between *v* and k(v), $E_{b}\left(v\right)$. Combined with Equation (4), we can also determine the effective elastic modulus of the lattice metastructured bar with different density distributions. Figure 11 shows the mapping relationships for the crisscrossed elliptical holes lattice. To improve the generality of the fitted model, a polynomial fit is applied, and the polynomial is expressed in terms of the parameters (a,b,c,d) for simplicity:

(44) $$a+bv+cv^{2}+dv^{3}.$$

### 4.3. Application of the VDPLM Under Tension

**Figure 12:**
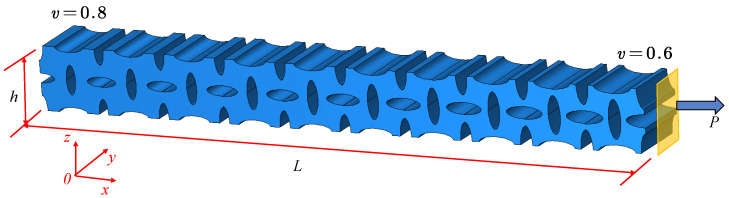
The variable-density lattice metastructure under tension.

In this section, the proposed surface-enabled computational homogenization method (SCHM) with the variable-density surface elastic model and the classical homogenization method (CHM) with the classical micromechanics homogenization model are applied to predict the size-dependent and density-dependent displacement of a VDPLM under a tensile force *P*. A high-fidelity FEM result is used as the reference to compare the error between the classical model and the variable-density surface elastic model, verifying their validity. The crisscrossed elliptical holes VDPLM, shown in Figure 12, is fixed at the left end, with a tensile force of P=1000N applied at the right end. The variation in the average displacement at the surface of the end is analyzed, considering changes in thickness, length, and density distribution.

The effective elastic modulus $\bar{E_{eff}}$ for the VDPLM of different lengths can be obtained by combining the mapping relations in Table 2 and the surface elastic model Equations (4) and (43). Then, from the stress–strain relationship,

(45) $$\bar{\varepsilon }=\frac{u}{L}=\frac{\bar{\sigma }}{\bar{E_{eff}}},$$

and the mean end displacement of the lattice metastructure can be obtained as

(46) $$u=L\frac{\bar{\sigma }}{\bar{E_{eff}}}=L\frac{P}{a^{2}}\frac{1}{\bar{E_{eff}}}.$$

Finally, the accuracy of the prediction results is verified by relative error:

(47) $$\delta =\frac{\left|u-u_{0}\right|}{u_{0}}\times 100\%.$$

**Figure 13:**
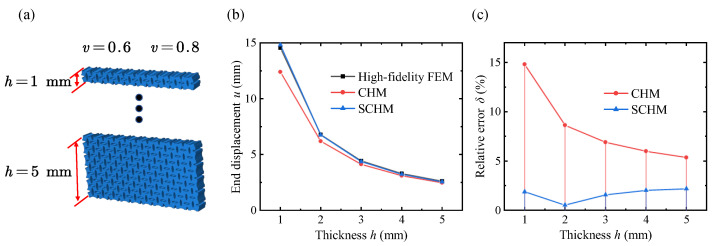
End displacement of the variable-density lattice metastructure with different thicknesses through the high-fidelity finite element method (high-fidelity FEM), classical homogenization method (CHM), and surface-enabled computational homogenization method (SCHM). (**a**) Variable-density lattice metastructure with different thicknesses. (**b**) End displacement results of different methods. (**c**) Relative error results of different methods.

The results of the mean end surface displacement at different thicknesses are shown in Figure 13. Both the classical homogenization model and the variable-density surface elastic model converge to the true value as the thickness increases. This is because the surface thickness remains constant while metastructure thickness decreases, leading to a weakening of the surface effect. The variable-density surface elastic model presented demonstrates higher prediction accuracy than the classical model across different thicknesses. The relative error ratio is 7.83 when the thickness is 1 mm.

**Figure 14:**
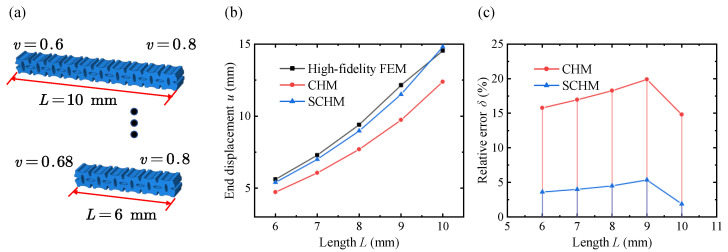
End displacement of the variable-density lattice metastructure with different lengths through the high-fidelity FEM, CHM, and SCHM. (**a**) Variable-density lattice metastructure with different lengths. (**b**) End displacement results of different methods. (**c**) Relative error results of different methods.

The end displacements at different lengths *L* are shown in Figure 14. The relative errors of the end displacements predicted by the variable-density surface elastic model are significantly smaller than those predicted by the classical homogenization model across the entire length range. The accuracy advantage of the surface elastic model becomes more pronounced as the length increases.

The lattice metastructure density distribution considered in the above analysis is linear. However, the variable-density surface elastic model imposes no limitations on the density distribution during its formulation. This demonstrates the versatility of the model for different density distribution modes. Next, the displacement predictions for each cross-section along the axial direction under various relative density distribution modes are analyzed.

**Figure 15:**
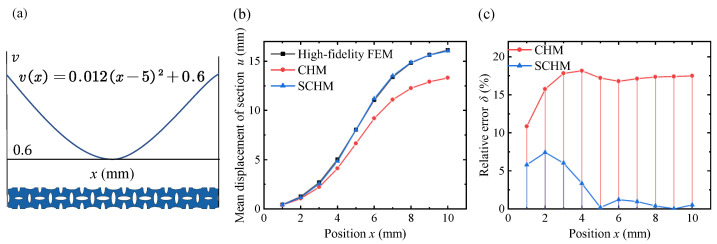
Displacement of each cross-section under the quadratic distribution of lattice density through the high-fidelity FEM, CHM, and SCHM. (**a**) Variable-density lattice metastructure under the quadratic distribution. (**b**) Mean displacement results of different methods. (**c**) Relative error results of different methods.

In the quadratic distribution mode, consider $v\left(x\right)=0.012{\left(x-5\right)}^{2}+0.6$ as an example, as shown in Figure 15. It can be observed that in the regions of higher relative density at both ends of the lattice metastructure, the lattice effective modulus is larger, and the surface effect is weaker. The displacement predicted by the classical homogenization model follows the same trend as the finite element results. However, in the region of lower relative density in the middle, the classical homogenization results deviate significantly from the finite element results due to the stronger surface effect. The relative error in the cross-section displacement prediction based on the variable-density surface elastic model is smaller than that of the classical homogenization model at all positions. As the distance from the cross-section to the fixed end increases, the relative error of the classical results gradually increases and stabilizes at a higher value, while the relative error of the variable-density surface elastic model increases initially and then decreases, maintaining a low level below 5%.

**Figure 16:**
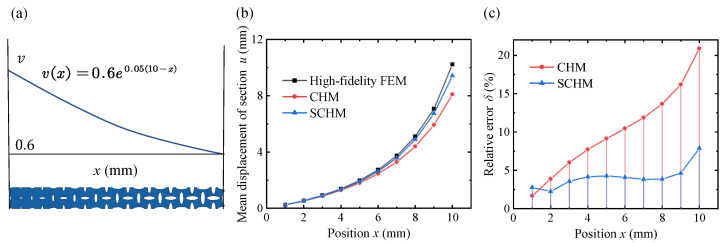
Displacement of each cross-section under the exponential distribution of the lattice density through the high-fidelity FEM, CHM, and SCHM. (**a**) Variable-density lattice metastructure under the exponential distribution. (**b**) Mean displacement results of different methods. (**c**) Relative error results of different methods.

In the exponential distribution mode, consider v(x)=0.6exp(0.05(10−x)) as an example, as shown in Figure 16. The relative density of the lattice decreases further from the fixed end, resulting in a smaller effective modulus and a stronger surface effect. From the relative error, it is obvious that the relative error of the classical homogenization model increases gradually, which is attributed to the accumulation of the error due to the surface effect. The relative error of the variable-density surface elastic model is consistently lower than that of the classical homogenization model, maintaining below 5%. The relative error of the variable-density surface elastic model is higher at positions x=1 and x=10 because the lattice at these positions cannot fully satisfy the scale separation assumption in the *x*-direction. However, the external feature length in the x-direction is much larger than the lattice constant, and the proportion of lattice that does not satisfy the scale separation assumption is very small, so its effect can be neglected.

**Figure 17:**
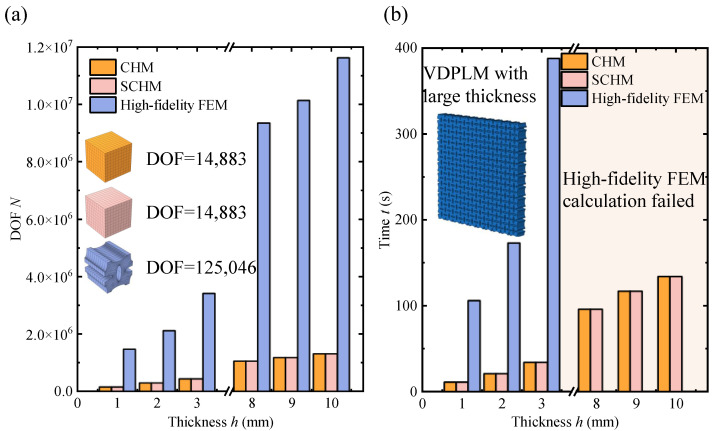
Computational DOF and efficiency of the high-fidelity FEM, CHM, and SCHM. (**a**) Computational DOF for different methods applied to the unit cell size and VDPLM with different thicknesses. (**b**) Computation time of the VDPLM with different thicknesses through different methods

Figure 17 shows the typical mesh, computational degrees of freedom, and computational time for each method, respectively. Compared to the high-fidelity FEM, the SCHM clearly requires fewer degrees of freedom and less computational time, similar to the CHM. For the case shown in Figure 13, when the thickness is 3mm, the computation time of the FEM is 11.4 times longer than that of the SCHM. As the thickness increases, the FEM becomes infeasible due to high memory consumption, whereas the SCHM continues to provide results with minimal computing resource consumption.

In summary, the SCHM significantly enhances computational efficiency and reduces resource consumption while maintaining high accuracy comparable to the FEM. This improves the applicability of the surface elastic model. Moreover, the method offers an obvious advantage in computational accuracy over the classical homogenization method.

## 5. Conclusions

This paper made two scientific and methodological contributions to the physical understanding and computational homogenization modeling of variable-density polymer lattice metastructures. First, we revealed a novel variable-density surface law governing the surface intrinsic length at macroscopic scales, which is fundamentally determined by lattice configuration. Second, a surface effect-enhanced computational homogenization framework was proposed that can capture the surface-induced size effects of variable-density polymer lattice metastructures. The main conclusions are summarized as follows:

(1) Compared with the bulk lattices, the lattices in the surface region lack the interaction of adjacent lattices. Therefore, the effective elastic modulus of lattices in the surface region is different from that in the bulk region, which is known as the surface effect. The varying volume occupancy and elasticity of these two regions contribute to the size-dependent effective elasticity of the lattice metastructure. In the VDPLM, the lattice density is position-dependent, which in turn causes the effective elasticity to also vary with position. Therefore, the VDPLM shows microstructure-dependent variable-density surface effects.
(2) To predict the mechanical properties of the VDPLM with high accuracy and efficiency, an FEM-based homogenization method for the VDPLM incorporating surface effect was proposed. An offline database of effective elastic modulus was established through the proposed method. According to the offline database, we revealed the variable-density surface law of the surface intrinsic length. The variable-density surface law shows that the surface intrinsic length is related to the lattice type, relative density, and lattice constant, where it shows a clear linear relationship with the lattice constant.
(3) An efficient and high-precision surface elastic model was proposed, which overcomes the inaccuracy of the classical model by inducing a surface intrinsic length obeying the variable-density surface law and the time-consuming problem of high-fidelity finite element analysis.

In summary, this innovative approach preserves critical configuration-dependent surface effects while achieving both efficiency and precision in predicting the macro-scale mechanical performance of the VDPLM.

## Figures and Tables

**Table 1:** Advantages and limitations of different homogenization methods.

Homogenization Methods	Advantages	Limitations
Rule of mixture	Simple model	Only the volume fraction is considered, not the specific geometry
Voigt–Reuss model	A simple manner to provide a reasonable estimate of the effective performance range	The range is too wide and is mainly used to test the correctness
Self-consistent method	Suitable for high volume fraction inclusions	Difficult to solve and usually no analytical solution can be obtained
Mori–Tanaka method	Simple calculation, suitable for low volume dispersed inclusions	Difficult to handle complex microstructures
Asymptotic homogenization method	Provides effective overall properties as well as local stress and strain values	The calculation is complicated
Method of cells	Computational efficiency is greatly improved	A loss of local field accuracy
Voronoi cell finite element method	Reduce the number of finite elements and the difficulty of calculation	Irregular finite element shapes, which increase errors

**Table 2:** Mapping relationships between *v* and k(v), and $E_{b}\left(v\right)$ of different lattices

Lattice Type	Mapping	Lattice Unit
Crisscrossed elliptical holes	$E_{b}\left(v\right)=\left(5.14,-24,31.58,-8.02\right)$	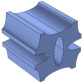
	$k\left(v\right)=\left(0.23,-1.856,3.013,-1.391\right)$	
Two-dimensional re-entrant hexagonal honeycomb in *x*	$E_{b}\left(v\right)=\left(-0.005,0.103,-0.728,2.475\right)$	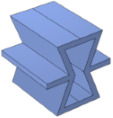
	$k\left(v\right)=\left(-0.295,0.098,-0.307,0.909\right)$	
Two-dimensional re-entrant hexagonal honeycomb in *z*	$E_{b}\left(v\right)=\left(-0.015,0.353,-2.805,12.188\right)$	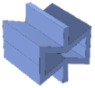
	$k\left(v\right)=\left(-0.001,0.022,-0.109,0.169\right)$	
Square honeycomb rotated	$E_{b}\left(v\right)=\left(-0.019,0.101,-0.134,2.604\right)$	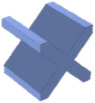
	$k\left(v\right)=\left(0.147,-1.748,4.765,-4.291\right)$	
Body centered cubic	$E_{b}\left(v\right)=\left(1.299,-6.31,9.94,-0.093\right)$	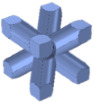
	$k\left(v\right)=\left(-0.02,-0.274,0.564,-0.264\right)$	
Body centered cubic foam	$E_{b}\left(v\right)=\left(-0.398,4.919,-5.459,5.627\right)$	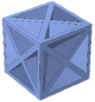
	$k\left(v\right)=\left(0.005,-0.159,0.337,-0.194\right)$	
Cube	$E_{b}\left(v\right)=\left(-0.117,2.845,-1.176,3.237\right)$	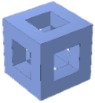
	$k\left(v\right)=\left(-0.006,0.001,-0.058,0.071\right)$	
Cubic plate lattice	$E_{b}\left(v\right)=\left(-0.042,3.803,-0.638,1.476\right)$	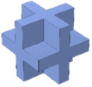
	$k\left(v\right)=\left(-0.012,0.009,0.003,-0.001\right)$	
Fisher–KochS	$E_{b}\left(v\right)=\left(-0.323,2.655,-2.672,5.352\right)$	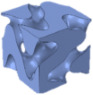
	$k\left(v\right)=\left(-0.213,0.731,-0.949,0.447\right)$	
Gyroid	$E_{b}\left(v\right)=\left(-0.05,0.469,2.262,1.969\right)$	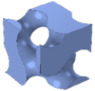
	$k\left(v\right)=\left(-0.714,3.72,-6.482,3.656\right)$	
I-WP	$E_{b}\left(v\right)=\left(0.052,-1.145,5.635,0.298\right)$	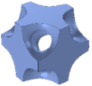
	$k\left(v\right)=\left(0.062,-0.669,1.332,-0.772\right)$	
Diamond	$E_{b}\left(v\right)=\left(-0.136,1.649,-2.422,6.483\right)$	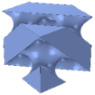
	$k\left(v\right)=\left(-0.353,1.385,-2.79,1.915\right)$	

## Data Availability

The original contributions presented in this study are included in the article. Further inquiries can be directed to the corresponding author.
